# Effects of pre-pregnancy body mass index and gestational weight gain on maternal and infant complications

**DOI:** 10.1186/s12884-020-03071-y

**Published:** 2020-07-06

**Authors:** Yin Sun, Zhongzhou Shen, Yongle Zhan, Yawen Wang, Shuai Ma, Suhan Zhang, Juntao Liu, Sansan Wu, Yahui Feng, Yunli Chen, Shuya Cai, Yingjie Shi, Liangkun Ma, Yu Jiang

**Affiliations:** 1 Department of Obstetrics and Gynecology, Peking Union Medical College Hospital, No. 1 Shuaifuyuan Wangfujing, Dongcheng District, Beijing, 100730 China; 2 Department of Epidemiology and Health Statistics, School of Public Health, Chinese Academy of Medical Sciences and Peking Union Medical College, No. 9 Dongdan Santiao, Dongcheng District, Beijing, 100730 China

**Keywords:** Chinese pregnant women, Gestational weight gain, Cohort study, Pre-pregnancy BMI, Maternal outcomes, Neonatal outcomes

## Abstract

**Background:**

The potential effects of pre-pregnancy body mass (BMI) and gestational weight gain (GWG) on pregnancy outcomes remain unclear. Thus, we investigated socio-demographic characteristics that affect pre-pregnancy BMIs and GWG and the effects of pre-pregnancy BMI and GWG on Chinese maternal and infant complications.

**Methods:**

3172 women were enrolled in the Chinese Pregnant Women Cohort Study-Peking Union Medical College from July 25, 2017 to July 24, 2018, whose babies were delivered before December 31, 2018. Regression analysis was employed to evaluate the socio-demographic characteristics affecting pre-pregnancy BMI and GWG values and their effects on adverse maternal and infant complications.

**Results:**

Multivariate logistic regression analysis revealed that age groups < 20 years (OR: 1.97), 25–30 years (OR: 1.66), 30–35 years (OR: 2.24), 35–40 years (OR: 3.90) and ≥ 40 years (OR: 3.33) as well as elementary school or education below (OR: 3.53), middle school (OR: 1.53), high school (OR: 1.40), and living in the north (OR: 1.37) were risk factors in maintaining a normal pre-pregnancy BMI. An age range of 30–35 years (OR: 0.76), living in the north (OR: 1.32) and race of ethnic minorities (OR: 1.51) were factors affecting GWG. Overweight (OR: 2.01) and inadequate GWG (OR: 1.60) were risk factors for gestational diabetes mellitus (GDM). Overweight (OR: 2.80) and obesity (OR: 5.42) were risk factors for gestational hypertension (GHp). Overweight (OR: 1.92), obesity (OR: 2.48) and excessive GWG (OR: 1.95) were risk factors for macrosomia. Overweight and excessive GWG were risk factors for a large gestational age (LGA) and inadequate GWG was a risk factor for low birth weights.

**Conclusions:**

Overweight and obesity before pregnancy and an excessive GWG are associated with a greater risk of developing GDM, GHp, macrosomia and LGA. The control of body weight before and during the course of pregnancy is recommended to decrease adverse pregnancy outcomes, especially in pregnant women aged < 20 or > 25 years old educated below university and college levels, for ethnic minorities and those women who live in the north of China.

**Trial registration:**

Registered at Clinical Trials (NCT03403543), September 29, 2017.

## Background

In recent years, the pre-pregnancy BMI of women of childbearing ages has shown an upward trend in developed countries [1]. The Pregnancy Risk Assessment Monitoring System (PRAMS) revealed that obesity prior to conception was as high as 22%, an increase of 69.3% compared with 10 years ago in the United States [1]. In China, the 2002 national nutrition survey revealed that being overweight (a BMI ≥ 24 kg/m^2^) and obese (a BMI ≥ 28 kg/m^2^) for women aged 18–44 reached 21.8 and 6.1%, respectively [2], and that there was an increasing trend particularly in women of childbearing age [3].

The nutritional status of mothers-to-be is believed to be a good predictor of perinatal and adverse long-term outcomes for both the infant and the mother [4]. Being overweight or obese before becoming pregnancy are high risk factors for GDM, hypertensive syndrome and disorders of fetal growth [5, 6]. In contrast, underweight pregnant women are at an increased risk of preterm birth (PB) and for delivering small-for-gestational-age (SGA) newborns [7, 8]. In addition, women who present with inadequate weight gain may experience complications such as anemia [9], PB [10], low birth weight (LBW) [11] and SGA [12], whereas women with excessive weight gain are more likely to develop GDM [13], GHp [14], preeclampsia [9] and the need for caesarean sections [15]. Therefore, it is of particular relevance to study the effects of pre-pregnancy BMI and GWG on pregnancy and the newborn, and to develop a reasonable pregnancy weight control plan. Most of the current evidence on pre-pregnancy BMI and GWG values comes from Western or high income countries [16].

The Chinese Pregnant Women Cohort Study-Peking Union Medical College (CPWCS-PUMC) is a multicenter, prospective and ongoing cohort study, which was established to provide relevant scientific evidence to guide the healthcare of pregnant Chinese women. In the present study, pregnant women from the CPWCS-PUMC in their first trimester were selected as subjects. We aimed to find the socio-demographic characteristics that could affect pre-pregnancy BMI and GWG values and the effects that these values may have on maternal and infant complications.

## Methods

### Study design and participants

This study was based on CPWCS-PUMC population research from 2017 to 2018 in 24 hospitals (secondary grades and above, with maternal and child health care centers accounting for two-thirds and general hospitals one-third of institutions) in 15 provinces (municipalities and autonomous regions) (Supplementary Figure 1). CPWCS-PUMC utilizes self-designed surveys for pregnant women and physicians. The pregnant women surveys consisted of four phases, namely the first trimester, the second trimester, the third trimester and the postpartum 6-week survey. Every survey included basic information and the status of physical care, environmental, physical activity, dietary and nutrient supplement, sleep, psychological, health and economic status. The physician-side survey was completed by physicians and epidemiologists and included three surveys about information on prenatal examination, maternal delivery outcomes and infant outcomes.

The inclusion criteria of the CPWCS-PUMC cohort study were: (1) Chinese nationality; (2) pregnancy ≤12 gestational weeks; (3) maternity files had been established in hospital; (4) regular birth inspection; (5) online completion of the survey; (6) signing of informed consent. Exclusion criteria were: (1) pregnancy > 12 gestational weeks; (2) those who could not have regular birth inspection; (3) floating population who did not live in the local area for a long time; (4) those who have contraindications to pregnancy such as gynecological tumors. In the present study, only single pregnancy outcomes were investigated.

Our local ethics committee approved the study (HS-1345) and all recruited women provided signed written consent forms.

### Recruitment

**Fig. 1:**
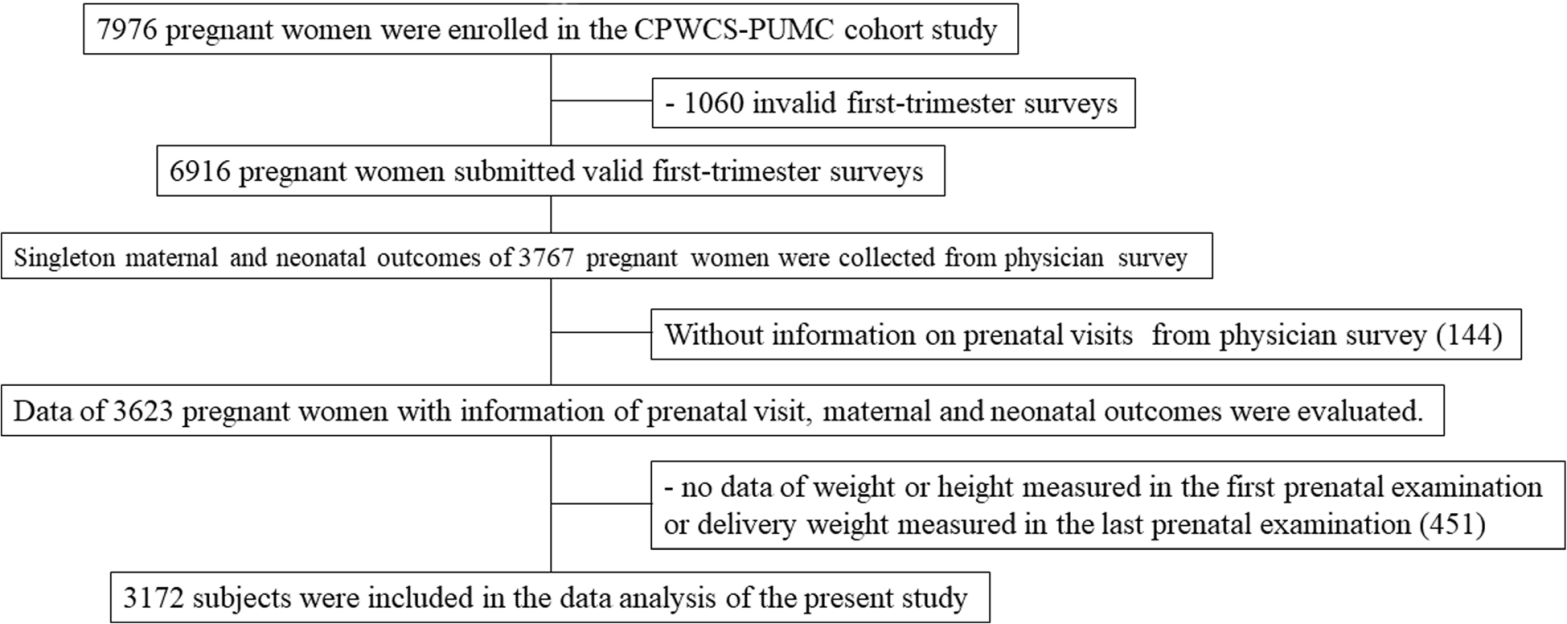
Flow chart of this study

From July 25, 2017 to July 24, 2018, 7976 pregnant women in the first trimester who met the inclusion criteria took part in the CPWCS-PUMC cohort study. A total of 6916 pregnant women submitted valid first-trimester surveys. Singleton maternal and neonatal outcomes of 3767 pregnant women with babies delivered before December 31, 2018 were collected from the physician survey. 144 pregnant women without information on prenatal visits from the physician survey were excluded. Data about 3623 pregnant women with information on prenatal examinations as well as maternal and neonatal outcomes were evaluated. A total of 451 pregnant women without weight or height data measured during the first prenatal examination or delivery weights measured at the last prenatal examination, were not eligible for inclusion. In total, 3172 pregnant women were included in the data analysis of the present study (Fig. 1**)**.

### Data collection

We collected socio-demographic data from pregnant women surveys including race, age, education level, living region, census register type, occupation, family member, self-income and family-income. We also measured the heights and weights of the women at their first prenatal examination, including their weights recorded at the last prenatal examination (data obtained from the prenatal examination information of the physician survey). Maternal outcomes from the physician survey including gestational weeks, delivery mode, maternal complications (e.g., anemia, premature membrane rupture, gestational diabetes mellitus and hypertension) were collected by physicians at the 6-week postpartum follow-ups. Neonatal outcomes from the physician survey including low and normal birth weights, macrosomia and small, normal or large size for gestational age (GA) were collected during physicians’ home visits to the mother’s home at the sixth week postpartum.

### Standard measurements

Physicians in the centers involved in the study collected anthropometric data. Mothers’ weights and heights were measured in light clothing but with no shoes on. Height was measured to the nearest 0.1 cm with a ruler and weight to the nearest 0.01 kg using calibrated electronic scales. Blood pressure was measured using a standard sphygmomanometer. The presiding physicians entered all relevant data into the hospital’s electronic medical records system.

BMI (kg/m^2^) values before pregnancy were calculated by measuring the height and weight of pregnant women at their first prenatal examination (pregnancy ≤12 gestational weeks). It is noteworthy that self-reported pre-pregnancy weights were highly correlated with those recorded at the initial prenatal visits [15]. BMI values before pregnancy were classified according to the WHO cut-off points for Asian adults [17, 18] (Supplementary Table 1). GWG refers to the difference between the weight measured at the last prenatal examination before delivery and the weight measured at the initial prenatal examination. GWG was classified following the 2009 Institute of Medicine (IOM) guidelines [19] (Supplementary Table 2).

GHp was defined as systolic blood pressure being ≥140 mmHg or diastolic pressure being ≥90 mmHg during the 3rd trimester, or if the mother-to-be had been prescribed medication to control hypertension [20]. GDM was diagnosed if one or more of the following criteria were met during pregnancy: fasting plasma glucose ≥5.1 mmol/L, 1 h plasma glucose levels ≥10.0 mmol/L, 2 h glucose levels ≥8.5 mmol/L after overnight fasting with a 75 g glucose load according to the WHO 2013 diagnostic criteria [21]. Prelabor rupture of membranes (PROM) was suspected based on symptoms and speculum examination and might have been supported by testing the vaginal fluid or by ultrasound [22]*.* Anemia in pregnancy was diagnosed as a hemoglobin (Hb) concentration < 110 g/L (11 g/dL) according to the WHO criteria [23].

The definition of macrosomia employed was a birth weight > 4000 g. A low birth weight was defined as < 2500 g, SGA as a birth weight < than the 10th percentile and LGA as a birth weight > than the 90th percentile for GA.

### Statistical analyses

Data were collated and analyzed using Microsoft Office Excel 2007 and SPSS Statistics for Windows (Version 25.0, IBM Corp, NY, US). The classification index describes the number and percentage of various types, and the chi-squared test or the exact probability method (if the chi-squared test was not appropriate) was employed for comparisons between groups. A cumulative logistic regression model was employed to correct the effect of confounding factors in order to analyze the socio-demographic characteristics affecting the BMI values before pregnancy and GWG. Multivariate logistic regression models (including cumulative logistic regression and multinomial logistic regression) were employed to correct for confounding factors permitting the analysis of independent risk factors for adverse outcomes for mothers maternal and neonates. A *P*-value < 0.05 was considered to be a significant finding.

## Results

### Socio-demographic characteristics affecting BMI values before pregnancy and GWG

Pre-pregnancy BMIs were classified into 4 types namely: underweight, normal, overweight and obese women (see Table 1**)**. There were significant differences in race (*P* <  0.001), age (*P* = 0.020), educational levels (*P* <  0.001), regions (*P* <  0.001), occupations (*P* = 0.030), self-income (*P* = 0.010) and family-income (*P* <  0.001) among the 4 pre-pregnancy BMI groups (Table 1). However, after correction by multivariate logistic regression analysis, it was found that in terms of age, compared with the 20–25 years age group, pregnant women of the age groups < 20 years old (OR: 1.97, *P* = 0.008), 25–30 years old (OR: 1.66, *P* <  0.001), 30–35 years old (OR: 2.24, *P* <  0.001), 35–40 years old (OR: 3.90, *P* <  0.001) and ≥ 40 years old (OR: 3.33, *P* <  0.001) were at risk to keep normal weight prior to pregnancy. From the view of education, compared with pregnant women with college or university degree, elementary school education and below (OR: 3.53, *P* = 0.006), middle school (OR: 1.53, *P* <  0.001) and high school (OR: 1.40, *P* = 0.001) were risk factors to keep normal pre-pregnancy BMI. From a regional perspective, pregnant women living in the south were more likely to control pre-pregnancy BMIs within the normal range than pregnant women in the north (OR: 1.37, *P* <  0.001; see Table 2**)**.

**Table 1:** Comparison of socio-demographic characteristics in the four pre-pregnancy BMI groups

	**Pre-pregnancy BMI category**				
	Underweight	Normal weight	Overweight	Obese	*P*-value
N (%)	420 (13.2)	2292 (72.3)	401 (12.6)	59 (1.9)	
**Race**					0.020
Han	401 (95.5)	2238 (97.6)	388 (96.8)	55 (93.2)	
Minorities	19 (4.5)	54 (2.4)	13 (3.2)	4 (6.8)	
**Age (years)**					< 0.001
< 20	20 (4.8)	69 (3.0)	12 (3.0)	5 (8.5)	
~ 20	69 (16.4)	213 (9.3)	34 (8.5)	2 (3.4)	
~ 25	224 (53.3)	1060 (46.2)	162 (40.4)	27 (45.8)	
~ 30	94 (22.4)	679 (29.6)	118 (29.4)	17 (28.8)	
~ 35	10 (2.4)	222 (9.7)	62 (15.5)	7 (11.9)	
≥ 40	3 (0.7)	49 (2.1)	13 (3.2)	1 (1.7)	
**Education level**					< 0.001
Elementary school or below	1 (0.2)	10 (0.4)	8 (2.0)	0 (0)	
Middle school	58 (13.8)	337 (14.7)	82 (20.4)	16 (27.1)	
High school	78 (18.6)	462 (20.2)	99 (24.7)	16 (27.1)	
College or university	255 (60.7)	1309 (57.1)	196 (48.9)	24 (40.7)	
Postgraduate degree	25 (6.0)	162 (7.1)	16 (4.0)	3 (5.1)	
PhD degree	3 (0.7)	12 (0.5)	0 (0)	0 (0)	
**Region**					< 0.001
South	188 (44.8)	820 (35.8)	120 (29.9)	15 (25.4)	
North	232 (55.2)	1472 (64.2)	281 (70.1)	44 (74.6)	
**Census register type**					0.565
Urban	177 (42.1)	982 (42.8)	167 (41.6)	20 (33.9)	
Rural	243 (57.9)	1310 (57.2)	234 (58.4)	39 (66.1)	
**Occupation**					0.030
Unemployed	123 (29.3)	647 (28.2)	139 (34.7)	20 (33.9)	
Managerial workers	41 (9.8)	236 (10.3)	39 (9.7)	3 (5.1)	
Professional and technical workers	64 (15.2)	372 (16.2)	58 (14.5)	10 (16.9)	
Clerical workers	48 (11.4)	236 (10.3)	22 (5.5)	6 (10.2)	
Merchant or service workers	76 (18.1)	449 (19.6)	77 (19.2)	5 (8.5)	
Farming or fishing workers	4 (1.0)	45 (2.0)	13 (3.2)	2 (3.4)	
Others	64 (15.2)	307 (13.4)	53 (13.2)	13 (22.0)	
**Family member**					0.087
1	3 (0.7)	30 (1.3)	4 (1.0)	0 (0)	
2	146 (34.8)	673 (29.4)	115 (28.7)	17 (28.8)	
3	86 (20.5)	519 (22.6)	93 (23.2)	8 (13.6)	
4	101 (24.0)	526 (22.9)	81 (20.2)	17 (28.8)	
5	59 (14.0)	409 (17.8)	73 (18.2)	9 (15.3)	
≥ 6	25 (6.0)	122 (5.3)	33 (8.2)	7 (11.9)	
Missing	0 (0)	13 (0.6)	2 (0.5)	1 (1.7)	
**Self-income (10,000 RMB (1410 USD)/year)**					0.010
≤ 1	53 (12.6)	284 (12.6)	71 (17.9)	13 (22.4)	
1–2	38 (9.0)	219 (9.7)	42 (10.6)	9 (15.5)	
2–4	114 (27.1)	607 (26.8)	104 (26.3)	23 (39.7)	
4–6	120 (28.6)	617 (27.3)	110 (27.8)	5 (8.6)	
6–8	33 (7.9)	170 (7.5)	22 (5.6)	2 (3.4)	
8–10	40 (9.5)	239 (10.6)	32 (8.1)	2 (3.4)	
> 10	22 (5.2)	126 (5.6)	15 (3.8)	4 (6.9)	
**Family-income (10,000 RMB (1410 USD) /year)**					< 0.001
≤ 10	223 (53.2)	1249 (55.4)	261 (65.9)	42 (73.7)	
10–20	144 (34.4)	719 (31.9)	97 (24.5)	10 (17.5)	
> 20	52 (12.4)	288 (12.8)	38 (9.6)	5 (8.8)	

Note: *RMB* Renminbi, *USD* US dollar

**Table 2:** Multivariate analysis of socio-demographic factors affecting pre-pregnancy BMI values

	OR (95% CI)	*P*-value
< 20 vs 20 ~ 25	1.97 (1.20–3.25)	0.008
25 ~ 30 vs 20 ~ 25	1.66 (1.26–2.19)	< 0.001
30 ~ 35 vs 20 ~ 25	2.24 (1.67–2.99)	< 0.001
35 ~ 40 vs 20 ~ 25	3.90 (2.75–5.54)	< 0.001
≥ 40 vs 20 ~ 25	3.33 (1.87–5.92)	< 0.001
Elementary school or below vs college or university	3.53 (1.43–8.75)	0.006
Middle school vs college or university	1.53 (1.22–1.92)	< 0.001
High school vs college or university	1.40 (1.15–1.72)	0.001
Postgraduate degree vs college or university	0.84 (0.61–1.16)	0.299
PhD degree vs college or university	0.34 (0.12–1.02)	0.054
North vs South	1.37 (1.16–1.62)	< 0.001

GWG was classified into 3 types (inadequate, adequate and excessive) according to IOM recommended criteria in Table 3. There were significant differences in age (*P* = 0.004), region (*P* = 0.002), census register type (*P* = 0.041) and family-income (*P* = 0.028) among the 3 GWG groups (Table 3). However, after correction by multivariate logistic regression analysis, it was found that compared with the age group range 20–25 years, 30–35 years old (OR: 0.76, *P* = 0.022) was a protective factor in gaining adequate weight during pregnancy. On the other hand, pregnant women living in the north (OR: 1.32, *P* <  0.001) and pregnant women of ethnic minorities (OR: 1.51, *P* = 0.041) were risk factors for gaining adequate weight during pregnancy (Table 4).

**Table 3:** Comparison of socio-demographic characteristics in the 3 GWG groups according to IOM recommendations

	Gestational weight gain category			
	Inadequate	Adequate	Excessive	*P*-value
N (%)	787 (24.8)	1309 (41.3)	1076 (33.9)	
**Race**				0.060
Han	773 (98.2)	1272 (97.2)	1037 (96.4)	
Minorities	14 (1.8)	37 (2.8)	39 (3.6)	
**Age (years)**				0.004
< 20	23 (2.9)	44 (3.4)	39 (3.6)	
~ 20	77 (9.8)	127 (9.7)	114 (10.6)	
~ 25	317 (40.3)	616 (47.1)	540 (50.2)	
~ 30	266 (33.8)	370 (28.3)	272 (25.3)	
~ 35	84 (10.7)	126 (9.6)	91 (8.5)	
≥ 40	20 (2.5)	26 (2.0)	20 (1.9)	
**Education level**				0.267
Elementary school or below	3 (0.4)	10 (0.8)	6 (0.6)	
Middle school	122 (15.5)	201 (15.4)	170 (15.8)	
High school	170 (21.6)	247 (18.9)	238 (22.1)	
College or university	430 (54.6)	749 (57.2)	605 (56.2)	
Postgraduate degree	58 (7.4)	94 (7.2)	54 (5.0)	
Ph.D. degree	4 (0.5)	8 (0.6)	3 (0.3)	
**Region**				0.002
South	318 (40.4)	476 (36.4)	349 (32.4)	
North	469 (59.6)	833 (63.6)	727 (67.6)	
**Census register type**				0.041
Urban	352 (44.7)	570 (43.5)	424 (39.4)	
Rural	435 (55.3)	739 (56.5)	652 (60.6)	
**Occupation**				0.134
Unemployed	231 (29.4)	358 (27.3)	340 (31.6)	
Managerial workers	83 (10.5)	135 (10.3)	101 (9.4)	
Professional and technical workers	124 (15.8)	223 (17)	157 (14.6)	
Clerical workers	75 (9.5)	139 (10.6)	98 (9.1)	
Merchant or service workers	145 (18.4)	252 (19.3)	210 (19.5)	
Farming or fishing workers	22 (2.8)	15 (1.1)	27 (2.5)	
Others	107 (13.6)	187 (14.3)	143 (13.3)	
**Family member**				0.215
1	8 (1.0)	11 (0.8)	18 (1.7)	
2	221 (28.1)	393 (30.0)	337 (31.3)	
3	176 (22.4)	290 (22.2)	240 (22.3)	
4	168 (21.3)	317 (24.2)	240 (22.3)	
5	158 (20.1)	217 (16.6)	175 (16.3)	
≥ 6	53 (6.7)	75 (5.7)	59 (5.5)	
Missing	3 (0.4)	6 (0.5)	7 (0.7)	
**Self-income (10,000 RMB (1410 USD)/year)**				0.294
≤ 1	118 (15.3)	155 (11.9)	148 (13.9)	
1–2	70 (9.1)	120 (9.2)	118 (11.1)	
2–4	196 (25.4)	349 (26.8)	303 (28.5)	
4–6	211 (27.3)	365 (28.1)	276 (26)	
6–8	55 (7.1)	96 (7.4)	76 (7.1)	
8–10	80 (10.3)	138 (10.6)	95 (8.9)	
> 10	43 (5.6)	77 (5.9)	47 (4.4)	
**Family-income (10,000 RMB (1410 USD) /year)**				0.028
≤ 10	410 (53.2)	725 (55.9)	640 (60.4)	
10–20	254 (32.9)	411 (31.7)	305 (28.8)	
> 20	107 (13.9)	162 (12.5)	114 (10.8)	

**Note:***RMB* Renminbi, *USD* US dollar

**Table 4:** Multivariate analysis of socio-demographic factors affecting GWG

	OR (95% CI)	*P*-value
< 20 vs 20 ~ 25	1.27 (0.84–1.92)	0.263
25 ~ 30 vs 20 ~ 25	1.09 (0.87–1.36)	0.467
30 ~ 35 vs 20 ~ 25	0.76 (0.60–0.96)	0.022
35 ~ 40 vs 20 ~ 25	0.79 (0.59–1.06)	0.112
≥ 40 vs 20 ~ 25	0.73 (0.45–1.19)	0.211
North vs South	1.32 (1.15–1.52)	< 0.001
Minorities vs Han	1.51 (1.02–2.24)	0.041

### Effect of pre-pregnancy BMI values on maternal and infant complications

In maternal outcomes, there were significant differences in the delivery mode, GDM and GHp (all *P* <  0.001) among the 4 pre-pregnancy BMI groups. For neonatal outcomes, there were significant differences in birth weights (and GA (both *P* <  0.001) among the 4 pre-pregnancy BMI groups (Table 5). After adjusting for the effects of confounding factors using a multivariate logistic regression model, we found that odd ratios in overweight pregnant women were 2.01 times and 2.80 times higher to suffer GDM (*P* <  0.001) and GHp (*P* <  0.001), and 1.92 times and 1.73 times higher to deliver macrosomia (*P* <  0.001) and LGA (*P* <  0.001) compared to normal weight pregnant women. Similarly, odd ratios in obese pregnant women were 5.42 times higher to suffer GHp (*P* <  0.001) and 2.48 times higher to deliver macrosomia (*P* = 0.019) compared to normal weight pregnant women (Table 7).

**Table 5:** Comparison of maternal and neonatal outcomes in the four pre-pregnancy BMI groups

	Pre-pregnancy BMI category				
	Underweight (*N* = 420)	Normal weight (*N* = 2292)	Overweight (*N* = 401)	Obese (*N* = 59)	*P*-value
Maternal outcomes					
**Gestational weeks**					0.267
≥ 28 and < 37	23 (5.5)	96 (4.2)	24 (6.0)	4 (6.8)	
≥ 37 and < 42	397 (94.5)	2191 (95.6)	377 (94.0)	55 (93.2)	
≥ 42	0 (0)	5 (0.2)	0 (0)	0 (0)	
**Delivery mode**					< 0.001
Eutocia	284 (67.6)	1312 (57.2)	172 (42.9)	26 (44.1)	
Caesarean section	126 (30.0)	948 (41.4)	223 (55.6)	32 (54.2)	
Midwifery practice	10 (2.4)	32 (1.4)	6 (1.5)	1 (1.7)	
**Maternal complications**					
**Anemia**					0.274
No	370 (88.1)	1970 (86.0)	336 (83.8)	53 (89.8)	
Yes	50 (11.9)	322 (14.0)	65 (16.2)	6 (10.2)	
**PROM**					0.132
No	373 (88.8)	2007 (87.6)	339 (84.5)	55 (93.2)	
Yes	47 (11.2)	285 (12.4)	62 (15.5)	4 (6.8)	
**GDM**					< 0.001
No	387 (92.1)	2041 (89)	325 (81)	47 (79.7)	
Yes	33 (7.9)	251 (11.0)	76 (19.0)	12 (20.3)	
**GHp**					< 0.001
No	415 (98.8)	2246 (98.0)	375 (93.5)	52 (88.1)	
Yes	5 (1.2)	46 (2.0)	26 (6.5)	7 (11.9)	
Neonatal outcomes					
**Birth weight**					< 0.001
Low birth weight	21 (5.0)	78 (3.4)	10 (2.5)	3 (5.1)	
Normal birth weight	383 (91.2)	2070 (90.3)	337 (84.0)	47 (79.7)	
Macrosomia	16 (3.8)	144 (6.3)	54 (13.5)	9 (15.3)	
**Gestational age (GA)**					< 0.001
Small for GA	32 (7.6)	137 (6.0)	13 (3.2)	0 (0)	
Normal GA	363 (86.4)	1934 (84.4)	311 (77.6)	48 (81.4)	
Large for GA	25 (6.0)	221 (9.6)	77 (19.2)	11 (18.6)	

Note: *PROM* Premature rupture of membranes, *GDM* Gestational diabetes mellitus, *GHp* Gestational hypertension

### Effect of GWG on maternal and infant complications

For maternal outcomes, there were significant differences in gestational weeks, delivery mode and GDM (all *P* <  0.001) and GHp (*P* = 0.004) among the 3 GWG groups. For neonatal outcomes, there were significant differences in birth weights and GA (both *P* <  0.001) in the 3 GWG groups (Table 6). After adjusting for the effects of confounding factors using a multivariate logistic regression model, we found that women who gained weight in the inadequate group had a 1.60 times higher odd ratio to suffer GDM (*P* <  0.001) and a 1.66 times higher odd ratios to give birth to weight babies (*P* = 0.022) compared to adequate weight gain women. Pregnant women who exhibited excessive weight gain had a 1.95 times higher odd ratio to deliver macrosomia (*P* <  0.001), and a 1.89 times higher odd ratios of delivering LGA (*P* <  0.001) compared to adequate weight gain pregnant women (Table 7).

**Table 6:** Comparison of maternal and neonatal outcomes in the three GWG groups according to IOM recommendations

	Gestational weight gain category			
	Inadequate (*N* = 787)	Adequate (*N* = 1309)	Excessive (*N* = 1076)	*P*-value
Maternal outcomes				
**Gestational weeks**				< 0.001
≥ 28 and < 37	61 (7.8)	53 (4.1)	33 (3.1)	
≥ 37 and < 42	724 (92.0)	1254 (95.8)	1042 (96.8)	
≥ 42	2 (0.2)	2 (0.1)	1 (0.1)	
**Delivery mode**				< 0.001
Eutocia	487 (61.9)	761 (58.1)	546 (50.7)	
Caesarean section	285 (36.2)	528 (40.3)	516 (48)	
Midwifery practice	15 (1.9)	20 (1.5)	14 (1.3)	
**Maternal complications**				
**Anemia**				0.695
No	681 (86.5)	1118 (85.4)	930 (86.4)	
Yes	106 (13.5)	191 (14.6)	146 (13.6)	
**PROM**				0.286
No	680 (86.4)	1159 (88.5)	935 (86.9)	
Yes	107 (13.6)	150 (11.5)	141 (13.1)	
**GDM**				< 0.001
No	665 (84.5)	1170 (89.4)	965 (89.7)	
Yes	122 (15.5)	139 (10.6)	111 (10.3)	
**GHp**				0.004
No	775 (98.5)	1279 (97.7)	1034 (96.1)	
Yes	12 (1.5)	30 (2.3)	42 (3.9)	
Neonatal outcomes				
**Birth weight**				< 0.001
Low birth weight	45 (5.7)	44 (3.4)	23 (2.1)	
Normal birth weight	711 (90.3)	1193 (91.1)	933 (86.7)	
Macrosomia	31 (3.9)	72 (5.5)	120 (11.2)	
**Gestational age (GA)**				< 0.001
Small for GA	60 (7.6)	78 (6.0)	44 (4.1)	
Normal GA	676 (85.9)	1119 (85.5)	861 (80.0)	
Large for GA	51 (6.5)	112 (8.6)	171 (15.9)	

Note: *PROM* Premature rupture of membranes, *GDM* Gestational diabetes mellitus, *GHp* Gestational hypertension

**Table 7:** Multivariate analysis of the effect of pre-pregnancy BMI and GWG on maternal and infant complications (All subjects adjusted for age, race, education level, family income)

	Pre-pregnancy BMI category			Gestational weight gain category	
	Underweight vs normal weight	Overweight vs normal weight	Obese vs normal weight	Inadequate vs adequate	Excessive v vs adequate
Gestational diabetes mellitus					
OR (95% CI)	0.80 (0.55–1.18)	2.01 (1.49–2.71)	1.91 (0.94–3.91)	1.60 (1.22–2.09)	0.90 (0.68–1.18)
*P*-value	0.263	**< 0.001**	0.075	**< 0.001**	0.437
Gestational hypertension					
OR (95% CI)	0.69 (0.27–1.78)	2.80 (1.67–4.69)	5.42 (2.26–13.03)	0.71 (0.36–1.41)	1.40 (0.85–2.29)
*P*-value	0.447	**< 0.001**	**< 0.001**	0.328	0.190
Low birth weight					
OR (95% CI)	1.48 (0.89–2.47)	1.00 (0.51–1.99)	2.30 (0.68–7.8)	1.66 (1.08–2.56)	0.70 (0.41–1.17)
*P*-value	0.128	0.997	0.181	**0.022**	0.171
Macrosomia					
OR (95% CI)	0.64 (0.37–1.09)	1.92 (1.35–2.72)	2.48 (1.16–5.29)	0.75 (0.48–1.16)	1.95 (1.43–2.67)
*P*-value	0.100	**< 0.001**	**0.019**	0.190	**< 0.001**
Small for GA					
OR (95% CI)	1.20 (0.8–1.81)	0.67 (0.37–1.21)	< 0.001 (< 0.001- > 999.9)	1.22 (0.86–1.75)	0.81 (0.55–1.18)
*P*-value	0.382	0.186	0.973	0.269	0.269
Large for GA					
OR (95% CI)	0.67 (0.44–1.04)	1.73 (1.28–2.33)	1.64 (0.82–3.27)	0.78 (0.55–1.11)	1.89 (1.45–2.46)
*P*-value	0.073	**< 0.001**	0.160	0.170	**< 0.001**

Note: *P*-values < 0.05 are highlighted in bold text; gestational age, GA

## Discussion

Through this survey, we found that, age, education level and region of China were factors that affected pre-pregnancy BMI, and that age, region and race were factors that affected GWG. For maternal and neonatal complications, being overweight and obese before pregnancy and unwarranted GWG were associated with an increased risk of GDM, GHp, macrosomia and LGA, and inadequate GWG bear greater risks for GDM and a low infant birth weight.

In our survey, it was established that pregnant women aged < 20 years and > 25 years old did not control pre-pregnancy BMIs within the normal range compared with the 20–25 year old age group. Studies have shown that too early and too late delivery increased the risk of adverse pregnancy outcomes [24–27] and also the risk of malformations [28, 29]. Therefore, women of these age groups should be recommended to control their weight within the normal range before pregnancy.

From the perspective of regional distribution, our result revealed that pregnant women living in the south were more likely to maintain normal pre-pregnancy BMIs and adequate GWG than pregnant women in the north, suggesting that the geographical location had an impact on these variables. Possible reasons may be that dietary culture varies between southern and northern regions, perhaps due to different climates and agricultural practices. A normal diet in the south typically involves a high intake of rice as staple food. In contrast, people in the north cultivate mainly wheat as their staple food [30]. Rice is a low-energy food containing about twice the quantity of water and about 50% of the energy compared with the same quantity of bread made from steamed wheat [31]. In addition, a colder climate in the north is associated with reduced physical activity and an energy-rich diet, factors likely to account for the measured increase in body weights [32].

For maternal complications, our study confirmed that being overweight before pregnancy was a risk factor for GDM, a result consistent with other recent findings [33–35]. GDM can seriously threaten the health of mothers and offspring [36]. Although the pathogenesis remains unclear, related studies have shown that insulin resistance is mainly caused by a series of physiological and pathological changes during pregnancy [37]. Adipose tissue is resistant to insulin action, resulting in lower levels of insulin receptors in fat [38, 39] and the number of insulin receptors in the body gradually decreases with increasing BMI. Therefore, regardless of pregnancy, individuals with BMIs have a greater risk of being diabetic than those with low BMIs. At the same time, due to physiological changes in the pattern of glucose metabolism during pregnancy, glucose tolerance is reduced [40], which further amplifies the risk of developing diabetes for pregnant women with high BMIs.

Being overweight and obese before pregnancy was proven to increase the risk of GHp in the present study. The possible mechanism is that has been weight increase leads to the accumulation of estrogen in the body due to the accumulation of fat. By mediating aldosterone secretion, sodium retention is caused by the renin angiotensin system or by directly increasing the recollection of the renal tubules, resulting in hypertension [41]. Furthermore, excessive fat accumulation can cause abnormal blood lipid metabolism, which is also related to gestational diabetes and hypertension [42]. Studies have shown that weight loss and control of obese pregnant women during pregnancy can reduce the risk of GHp (OR = 0.31; 95% CI: 0.11 ~ 0.84) [43].

The energy sources that mothers provide for fetal development include energy reserves before pregnancy and food acquisition during pregnancy [44]. The neonatal complications revealed in our study strongly indicated that being overweight and obese, and excessive GWG were important risk factors for macrosomia and LGA, while inadequate GWG was a risk factor for low birth weight, indicating that there is a clear correlation between maternal obesity and infant size at birth. The findings were consistent with other research results [45–47]. Being overweight and obese before pregnancy, and unacceptable weight gain during pregnancy may lead to increased concentrations of glucose, amino acids and free fatty acids in pregnant women [48], thereby increasing the risk of abnormal infant weight at birth. Therefore, pre-pregnancy BMI and GWG have similar roles in infant size. Research led by Tiffany et al. [49] showed that regardless of the pre-pregnancy body mass index, controlling weight gain during pregnancy is of great significance for reducing the risk of SGA and LGA. Therefore, it is of great importance to pay attention to pre-pregnancy BMIs and GWGs to ensure normal birth weights of newborns.

One strength of the present investigation was that data were collected from a large population-based cohort and that exposure and outcome measures were prospectively assessed. GWG was determined from authentic prenatal examination records instead of relying on memory recall at the end of pregnancy. However, several limitations should be taken into consideration. First, the sample size may still not be large enough for stratification, such as age, which may lack power for a robust assessment. Second, the pre-pregnancy weight and height were actually the weight and height measured during the initial prenatal examination and may therefore be biased. Moreover, measurement implementation and protocols for maternal anthropometry were standardized at the various study institutions, which may have biased the classification of the pre-pregnancy BMI. Third, there were no details about potential confounding factors such as clinical complications or lifestyle changes during pregnancy. Finally, there is no way to control completely pregnant women’s recall bias with regard to socio-demographic data.

## Conclusions

Overweight and obesity before pregnancy and excessive GWG were linked to an increased risk of GDM, GHp, macrosomia and LGA. In clinical practice, physicians can guide pregnant women to manage and control weight gain during pregnancy in order to reduce the risk of adverse pregnancy outcomes. Women of childbearing age can be advised on the importance of maintaining an optimal BMI when planning to become pregnant. Pregnant women aged < 20 or > 25 years old, with an education level below university and college, the race of ethnic minorities and living in the north should be given particular guidance on perinatal health-related knowledge and necessary interventions during the perinatal care process.

## Supplementary information

**Additional file 1: Supplementary Table 1.** Pre-pregnancy BMI categorization according to the WHO cut-points for Asian adults. **Supplementary Table 2.** Gestational weight gain (GWG) categorization according to the 2009 Institute of Medicine (IOM) recommendations.

**Additional file 2: Supplementary Figure 1.** CPWCS-PUMC project site distribution map.

## Data Availability

The datasets used and/or analyzed during the current study are available from the corresponding author on reasonable request.

## Abbreviations

BMI: Body mass index
CPWCS: Chinese Pregnant Women Cohort Study
CPWCS-PUMC: Chinese Pregnant Women Cohort Study-Peking Union Medical College
GA: Gestational age
GDM: Gestational diabetes mellitus
GHp: Gestational hypertension
GWG: Gestational weight gain
LBW: Low birth weight
PB: Preterm birth
PROM: Premature rupture of membranes
SGA: Small-for-gestational-age
USD: US dollar
