# Supplementary material for: Effects of pre-pregnancy body mass index and gestational weight gain on maternal and infant complications
Source: BMC Pregnancy Childbirth. 2020 Jul 6;20:390. doi: 10.1186/s12884-020-03071-y (PMC7336408; doi:10.1186/s12884-020-03071-y)
Supplement: Supplementary file 1 — Additional file 1: Supplementary Table 1. Pre-pregnancy BMI categorization according to the WHO cut-points for Asian adults. Supplementary Table 2. Gestational weight gain (GWG) categorization according to the 2009 Institute of Medicine (IOM) recommendations. [file 12884_2020_3071_MOESM1_ESM.docx]

**Supplementary Table. 1** Pre-pregnancy BMI categorization according to the WHO cut-points for Asian adults

|  | Underweight | Normal weight | Overweight | Obese |
| --- | --- | --- | --- | --- |
| Pre-pregnancy BMI in kg/m^2^ | < 18.5 | 18.5–24.9 | 25–29.9 | ≥ 30 |

**Supplementary Table 2.** Gestational weight gain (GWG) categorization according to the 2009 Institute of Medicine (IOM) recommendations

|  | Underweight | Normal weight | Overweight | Obese |
| --- | --- | --- | --- | --- |
| Inadequate | GWG < 12.5 kg | < 11.5 kg | < 7 kg | < 5 kg |
| Adequate | 12.5 ≤ GWG ≤ 18 kg | 11.5 ≤ GWG ≤ 16 kg | 7 ≤ GWG ≤ 11.5 kg | 5 ≤ GWG ≤ 9 kg |
| Excessive | GWG > 18 kg | >16 kg | > 11.5 kg | > 9 kg |
