# Supplementary figures and images for: Effects of pre-pregnancy body mass index and gestational weight gain on maternal and infant complications
Source: BMC Pregnancy Childbirth. 2020 Jul 6;20:390. doi: 10.1186/s12884-020-03071-y (PMC7336408; doi:10.1186/s12884-020-03071-y)

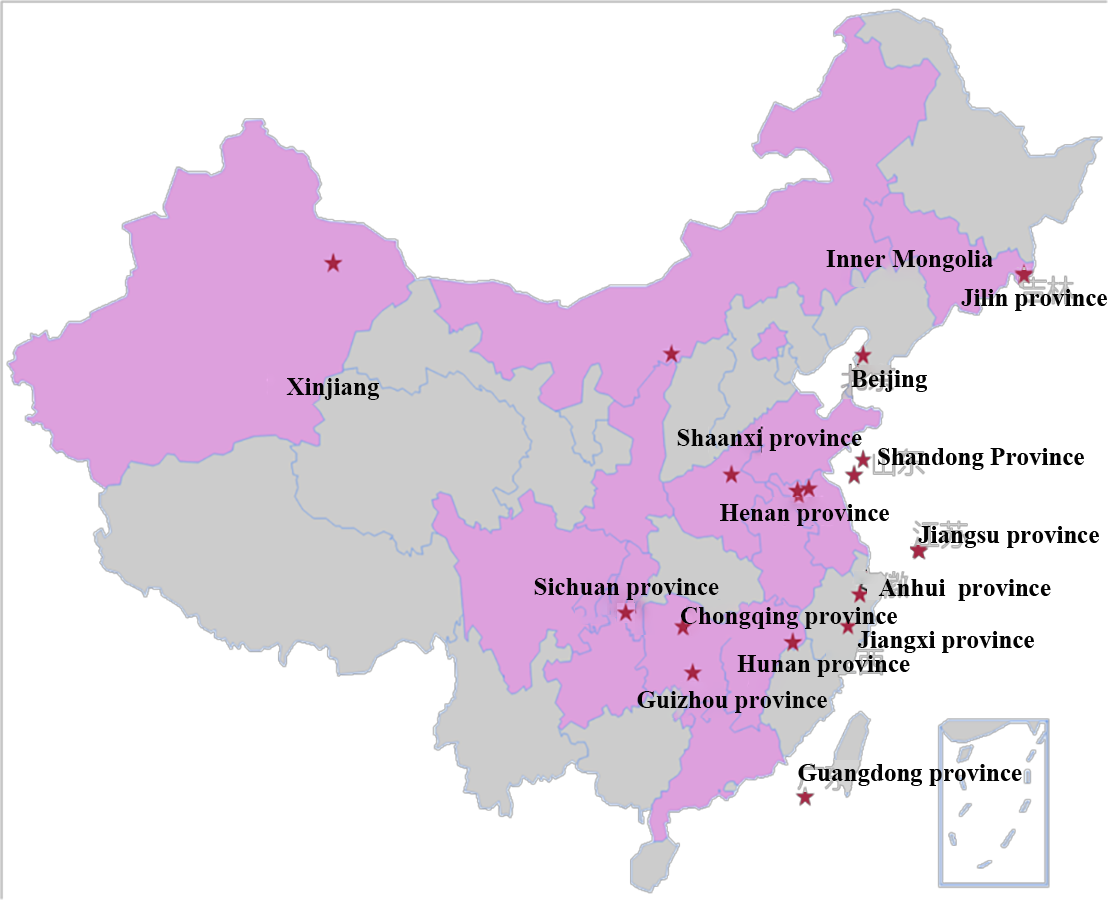

Supplement: Supplementary file 2 — Additional file 2: Supplementary Figure 1. CPWCS-PUMC project site distribution map. [file 12884_2020_3071_MOESM2_ESM.tif]
